# Noise Power Minimization in CMOS Brain-Chip Interfaces

**DOI:** 10.3390/bioengineering9020042

**Published:** 2022-01-18

**Authors:** Lorenzo Stevenazzi, Andrea Baschirotto, Giorgio Zanotto, Elia Arturo Vallicelli, Marcello De Matteis

**Affiliations:** 1 Department of Physics, University of Milano-Bicocca, Piazza della Scienza 3, 20126 Milano, Italy; andrea.baschirotto@unimib.it (A.B.); g.zanotto1@campus.unimib.it (G.Z.); marcello.dematteis@unimib.it (M.D.M.); 2 Section of Milano Bicocca, National Institute of Nuclear Physics, Piazza della Scienza 3, 20126 Milano, Italy; e.vallicelli@campus.unimib.it

**Keywords:** biological neural networks, biosensors, neural engineering, analog integrated circuits, low-noise amplifier

## Abstract

This paper presents specific noise minimization strategies to be adopted in silicon–cell interfaces. For this objective, a complete and general model for the analog processing of the signal coming from cell–silicon junctions is presented. This model will then be described at the level of the single stages and of the fundamental parameters that characterize them (bandwidth, gain and noise). Thanks to a few design equations, it will therefore be possible to simulate the behavior of a time-division multiplexed acquisition channel, including the most relevant parameters for signal processing, such as amplification (or power of the analog signal) and noise. This model has the undoubted advantage of being particularly simple to simulate and implement, while maintaining high accuracy in estimating the signal quality (i.e., the signal-to-noise ratio, SNR). Thanks to the simulation results of the model, it will be possible to set an optimal operating point for the front-end to minimize the artifacts introduced by the time-division multiplexing (TDM) scheme and to maximize the SNR at the a-to-d converter input. The proposed results provide an SNR of 12 dB at 10 µV_RMS_ of noise power and 50 µV_RMS_ of signal power (both evaluated at input of the analog front-end, AFE). This is particularly relevant for cell–silicon junctions because it demonstrates that it is possible to detect weak extracellular events (of the order of few µV_RMS_) without necessarily increasing the total amplification of the front-end (and, therefore, as a first approximation, the dissipated electrical power), while adopting a specific gain distribution through the acquisition chain.

## 1. Introduction

The most recent and relevant advances in neuroscience and, more specifically, in the analysis of the functioning of biological neural networks are directly proportional to the ability to observe by minimally invasive probing the weak extracellular neuro-potential signals generated by the electrical activity of specific nerve cell populations.

Today, planar micro-electrode arrays (MEAs) represent one of the most interesting devices for observing such cellular electrical events without penetrating the cell membrane [1,2,3].

They can be integrated on standard CMOS silicon dies and, therefore, be locally equipped with analog signal acquisition and processing circuits, the requirements of which are low power dissipation, high signal-to-noise ratio (SNR) and low area occupation [4].

To meet these requirements, most modern and efficient MEAs use signal acquisition schemes based on time-division multiplexing (TDM) algorithms [5,6,7].

This approach allows for implementation of a single acquisition channel for a certain set (array) of recording sites (electrodes/pixels), leveraging the low bandwidth of the neuro-potential signal (up to few kHz, and thus potentially easy to use in oversampling digitalization systems) and the processing speed of the CMOS circuits, while reducing the area and electrical power (i.e., the key aspects for efficiency and portability of next-generation MEAs).

**Figure 1:**
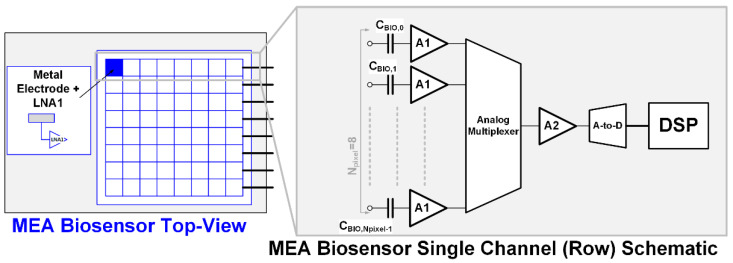
Block scheme of the single row of the micro-electrode array.

Figure 1 shows a simplified block scheme of a neural recording MEA with the highlight on the single channel scheme. They are typically arranged in a matrix of pixels, where each recording site (distributed in a single row) feeds a low-noise amplifier (LNA (A1), whose dc gain is G_1_) [8] by C_BIO,i_ (with i = 0 − N_pixel_ − 1) coupling capacitances. Each LNA drives the signal coming from a specific recording site. The outputs of these LNAs are then connected to a TDM scheme (by the analog multiplexer) that samples the signal coming from N_pixel_ (=8, in this case). Thus, the second (single) amplifier stage (A2, whose dc gain is G_2_) and the a-to-d converter can be simultaneously used for analog processing and digitalization of N_pixel_ = 8 recording sites, reducing the power and area of the whole biosensor.

Adopting a TDM architecture, thus integrating multiple parallel channels in a small silicon area, might lead to crosstalk: a signal originating from a specific pixel, which induces interference in the nearby channels due to the presence of parasitics. The wire width and their positions (i.e., horizontal adjacent wires in the same metal layer, or vertical adjacent ones in consecutive metal layers) determine the crosstalk coefficient [9]; therefore, a proper layout is necessary to limit this effect which, in this way, can be lowered below the intrinsic electronic noise. In addition to that, each LNA (corresponding to each electrode) has a different voltage offset at the output node (the node connected to the input of the TDM stage) due to an electrical properties mismatch involving integrated circuits MOS transistors (MOSTs) [10]. Such offsets mainly depend on MOST threshold voltage variation and cannot be rejected by classical ac-coupling capacitors because the TDM sampling stage converts such static voltage offset into dynamic (time-domain variant) artifacts [11,12,13], whose signal power is proportional to the LNA offset voltage standard deviation.

This leads to two important drawbacks as follows: If this effect has larger or comparable power than the neuro-potential signals at the input of the second amplifier stage, then it can saturate the input dynamic range of the a-to-d converter. If, on the other hand, it has lower power, it should decrease the output SNR and must be rejected using digitally assisted techniques that, in turn, require an extra power budget to be allocated to the digital signal processing (DSP) stages driven from the a-to-d converter.

This paper proposes an alternative design approach that, in principle, does not require an additional power budget for DSP and, at the same time, avoids any dynamic range issues for the a-to-d converter operation. The first step is to build an easy to use model based on simplified equations that fully include all electronic noise sources (thermal and flicker localized on electrode–LNA interface) and TDM artifacts. By simulating the model behavior vs. G_1_ (G_2_) gain at constant 60 dB gain for the whole chain (G_1_ + G_2_, where both gains are taken in dB), it is possible to find a threshold value of G_1_ at which the TDM artifacts become much smaller, and thus negligible, than the electronic noise power, maximizing in this way the a-to-d converter input SNR. 

The proposed paper is organized as follows: Section 2 presents the model, the signal processing block scheme, and main equations, respectively. Section 3 introduces the analytical expressions modelling the relevant noise sources in TDM MEAs schemes. In Section 4, the time-domain simulation results of the neural recording TDM channel are presented, setting the optimum operating point that maximizes the input dynamic range of the a-to-d converter and minimizes the total noise power, enhancing the channel SNR. At the end of the paper, conclusions will be drawn.

## 2. Time-Division Multiplexing Neural Recording Analog Front-End (Neural AFE)

The block scheme of the TDM neural recording analog front-end (neural AFE) under consideration is illustrated in Figure 1.

Each electrode is connected to a proper LNA, which has a gain G_1_ and a dominant pole, whose pulsation (frequency) is ω_1_ = 2·π·7.5 kHz, since most of the neural activity is in the (0.1 Hz, 5 kHz) bandwidth [14,15]. The main objectives of the first LNA are to amplify the weak extracellular signal coming from the planar electrode and to limit the acquisition bandwidth, avoiding any signal corruption due to the aliasing of the out-of-band electronic noise power spectral density (PSD) introduced by the TDM sampling operation. The LNA transfer function G_1_(s) is:

(1) $$G_{1}(s)=\frac{G_{1}}{{1+s/\omega }_{1}}$$

where G_1_ is the dc gain. The N_pixel_ = 8 signals coming from the electrode LNAs are then combined into a single transmission channel in different fixed-length time slots, adopting the TDM scheme. TDM operates at a frequency of f_TDM_ = 256 kHz, given by the following equation:

(2) $$f_{\mathrm{TDM}}=2\cdot f_{0,\mathrm{neuro}}\cdot N_{\mathrm{pixel}}\cdot \mathrm{OVR}$$

where f_0,neuro_ is the maximum frequency (1 kHz in this work) of the neuro-potential signal, N_pixel_ = 8 is the TDM pixel count and OVR (=16) is the oversampling ratio. OVR = 16 is required to effectively sample the neural spike for the DSP stage following the a-to-d converter and N_pixel_ = 8 is a tradeoff between the bandwidth and the number of second amplification stages in order to reduce power consumption [5,11,12,13,16]. Table 1 reports the model design parameters concerning the single channel/row analog signal processing.

The second amplification stage has a higher passband frequency range since the TDM operation modulates the signal into a larger bandwidth (128 kHz). The dc gain is G_2_ and the pulsation of the dominant pole is $\omega_{2}=2\pi \cdot 1\;\mathrm{MHz}$, which has been set higher than the input signal bandwidth in order to meet the settling time requirements of the signal in the 7.8125 µs (=1/128 kHz) multiplexing time width or period. The transfer function of the second amplifier is:

(3) $$G_{2}(s)=\frac{G_{2}}{{1+s/\omega }_{2}}$$

Choosing G_1_ (B) + G_2_ (dB) = 60 dB to amplify an extracellular neural signal of the order of tens of µV_RMS_ [17], and effectively digitalizing it, it is possible to consider different combinations of the individual gains to optimize the channel SNR.

When the single electrode signal is sampled, the channel has the following Laplace domain transfer function:

(4) $$G_{12}{(s)=G}_{1}\left(s\right)\cdot G_{2}(s)=\frac{G_{1}}{{1+s/\omega }_{1}}\cdot \frac{G_{2}}{{1+s/\omega }_{2}}$$

**Figure 2:**
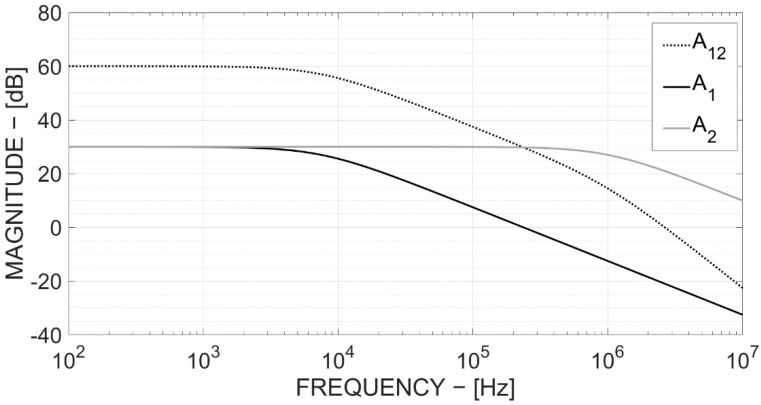
G_1_, G_2_ and G_12_ frequency response (with G_1_ = 30 dB and G_2_ = 30 dB).

Figure 2 shows the magnitude frequency response of both the first and second stage amplifiers (A1 and A2) when G_1_ = 30 dB and G_2_ = 30 dB, compared with the chain magnitude (A12). 

Finally, the a-to-d converter stage digitalizes the analog signal, which is later processed by the DSP (implementing communication and filtering of the input coming from the MEA channel and recovering the signals of the individual electrodes). 

Adopting the TDM scheme simplifies circuital architecture and reduces both the power and area of the channel. The count of amplifiers (after TDM) and a-to-d converters is decreased by a factor N_pixel_ − 1, where N_pixel_ is the count of multiplexed electrodes. However, the TDM modulates the voltage offset at the output LNAs, converting a static deviation (voltage offset) into a time-variant artifact, whose contribution to the whole channel SNR must be adequately considered.

## 3. Neural AFE Electronic Noise and TDM Artifact Sources

With reference to the single row of neural recording MEAs, the single channel has two main noise sources, electronic noise (v_ne_, whose PSD is <v^2^_ne_>/Δf) and a-to-d quantization noise (v_nADC_), and TDM-modulated artifacts (v_nTDM_, whose PSD is <v^2^_nTDM_>/Δf).

**Figure 3:**
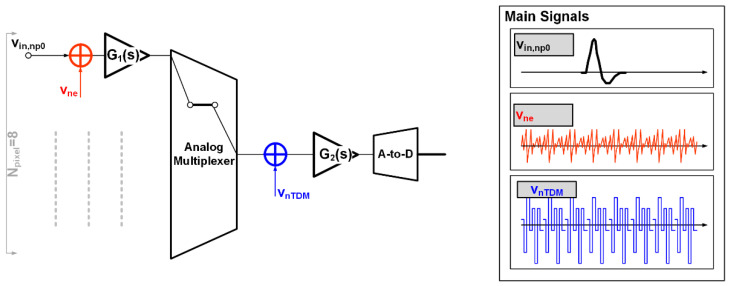
Model of the acquisition channel, showing only one electrode to highlight the noise and TDM artifact sources.

The acquisition channel model in Figure 3, including all relevant noise sources, introduces the electronic noise (v_ne_) at the beginning of the chain (where silicon circuits interface with the cells) and the TDM artifact source, which models the offset-sampling effect after the first amplification stage. Values are summarized in Table 2.

Assuming the electronic noise of the two amplification stages is of the same order, contributions from A2 are minor when input-referred. Similarly, the electronic noise of the a-to-d converter is neglected due to being divided by the acquisition channel gain when compared with the other noise sources at the interface. The input stage MOST in the first amplification stage (A1) is responsible for the main electronic noise PSD, and its contribution has two main components, flicker and thermal ones:

(5) $$\frac{{<v}_{N,\mathrm{MOS}}^{2}>}{\Delta f}=\frac{k_{F}}{f}+\frac{2}{3}\cdot 4\cdot k_{B}\cdot T\cdot \frac{1}{g_{\mathrm{ms}}}$$

where k_F_ is the flicker constant depending on the specific CMOS process technology and input MOST area, k_B_ is the Boltzmann constant (=13.8 10^−24^ J K^−1^), and T (=300 K) is the environment temperature. 

The first term in Equation (5) models flicker noise PSD, dominating at low frequencies. For this reason, it is important to reduce its influence by increasing the MOST area (reducing k_F_ value), although the requirement of large spatial resolution in planar MEA sets an upper limit for the same MOST area (for instance, approximately equal to 48 µm^2^ for this model where the MEA has 256 electrode/mm^2^). 

The second term in Equation (5) models thermal noise contribution, inversely proportional to MOST transconductance (g_ms_) and with a constant PSD. g_ms_ increases with MOST drain-source current at a limited power budget for portable implantable devices. State-of-the-art operates by lower than 10 µA [18,19,20,21] current consumption for the first amplification stage, resulting in approximately 50–100 µA/V.

Starting from these considerations, the obtained input-referred noise power over 1 Hz–5 kHz neuro-potential bandwidth is v_ne,RMS_ = 10 µV_RMS_, which also includes the minor contributions from all the other electronic components of the channel. 

**Figure 4:**
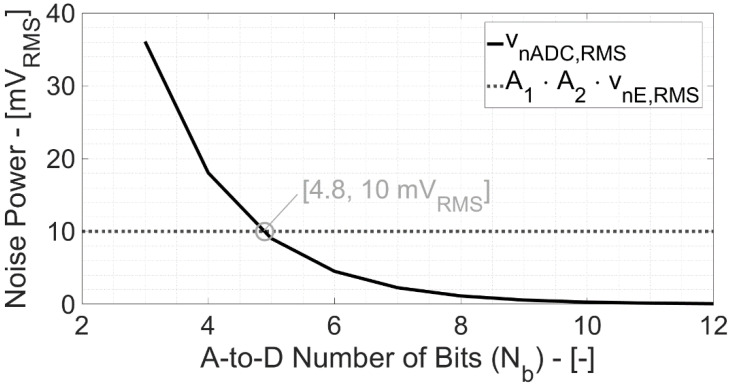
Quantization noise power of the a-to-d converter vs. number of bits (N_b_, compared to the electronic noise after 60 dB (A1·A2) amplification).

Figure 4 illustrates the quantization noise of a Nyquist a-to-d converter vs. number of bits. The quantization noise power is given by the well-known Equation:

(6) $$v_{\mathrm{nADC},\mathrm{RMS}}=\frac{\mathrm{FS}}{2^{\mathrm{Nb}}}\cdot \frac{1}{\sqrt{12}}$$

where FS (=1 V, in this model) is the analog input full-scale and N_b_ is the a-to-d converter number of bits. With N_b_ > 5, the quantization noise becomes lower than electronic one and then negligible for higher N_b_ values.

Each acquisition channel shows a different offset at the output node of the first amplification stage. Using N_pixel_ = 8 electrodes per channel, the dc value of the first amplification stage is then sampled by f_TDM_, resulting in undesired, high-frequency artifacts whose power is, in the first approximation, equal to such dc offset standard deviation (=300 µV_RMS_ in this model, corresponding to a threshold voltage standard deviation of 100 µm^2^ area MOST; for instance, a W/L aspect ratio of 100 µm/1 µm). 

## 4. Neural AFE Model Simulation Results

The presented model has been built starting from Equations (1) and (6) and is based on the block scheme in Figure 1 and Figure 3. It allows for an easily obtained (with very short simulation run times) realistic evaluation of the impact of the electronic noise and offset-sampling effect contributions of the chain on the channel SNR. More specifically, this paper adopts a MATLAB implementation of the neural AFE model. 

The first amplification stage introduces an electronic noise at the interface of the order of 10 µV_RMS_, assuming a current consumption of few µA (about 1.5 µA), in line with many implementations of MEA biosensors present in the literature [6,21,22]. On the other hand, it is more complicated to set the TDM artifact signal to the same values because it would involve using MOSTs with very large areas to minimize the A1 output voltage offset, induced by MOST threshold voltage standard deviation [10]. For instance, <10 µV threshold voltage standard deviation requires >0.09 mm^2^, MOST area (W·L), which, in turn, would imply MOST width (W) values equal to about 9 mm at MOST lengths (L) of 100 nm.

Basically, the electronic noise signal has a very small power, but it is amplified by two amplification stages (A1 and A2), while instead the TDM artifacts are amplified only by a factor G_2_ and have a power of more than an order of magnitude higher than the power of v_ne_. Therefore, for low G_1_ gain values, the offset-sampling effect will be dominant, whereas its contribution almost disappears (replaced by the electronic noise) at higher G_1_ values.

It is then of fundamental importance to evaluate how G_1_ and G_2_ gain values influence the channel SNR. The neural AFE channel has been simulated in the time domain (by sweeping G_1_ from 0 dB to 60 dB with 1 dB/step and maintaining G_1__2_ (dB) = G_1_ (dB) + G_2_ (dB) = 60 dB vinculum). Electronic noise (v_ne_) and TDM artifact (v_nTDM_) signals experience G_1__2_ (see Equation (4)) and G_2_ (see Equation (3)) amplification, respectively.

**Figure 5:**
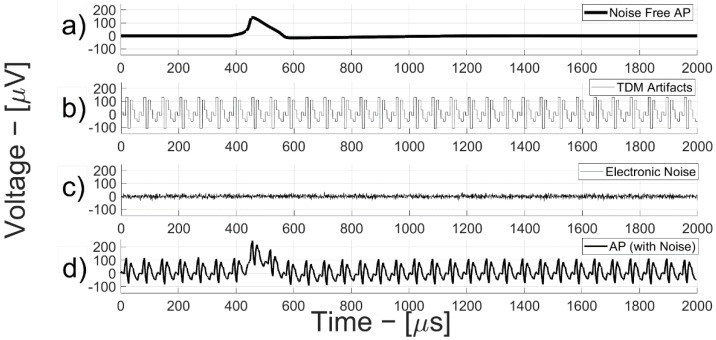
Action potential vs. time with noise and TDM artifact contributions. (**a**) noise-free AP; (**b**) TDM artifacts; (**c**) electronic noise; (**d**) neural AFE channel (AP, box).

Figure 5 shows the time-domain evolution of a classic action potential (AP) signal (0.2 ms time duration) processed by the neural AFE channel (AP, box (d)) by decomposing the contributions of the individual components as follows (Figure 5b–d). All the signals in Figure 5 are referred to the input node of the neural AFE model for a total chain gain of 60 dB, with 12 dB on the first stage gain (G_1_).

These simulations were obtained by assuming an AP signal at the neural AFE input of 100 µV_RMS_.

In these conditions, the TDM artifacts are evidently dominant and involve a significant loss of SNR compared to the ideal case of considering the only electronic noise. Two particularly critical effects are generated: an increase in the input dynamic range of the a-to-d converter (with a consequent increase in the dynamic power consumption) and a reduction of the SNR compared to the mere presence of electronic noise.

**Figure 6:**
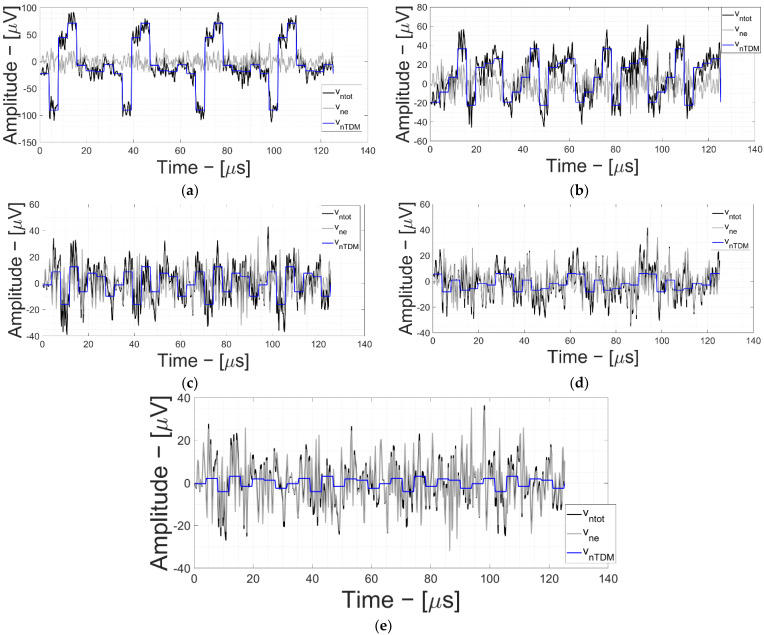
Neural AFE electronic noise (v_ne_) and TDM artifacts (v_nTDM_) time-domain simulations vs. G_1__._ (**a**) G_1_ = 18 dB and G_2_ = 42 dB; (**b**) G_1_ = 24 dB and G_2_ = 36 dB; (**c**) G_1_ = 30 dB and G_2_ = 30 dB; (**d**) G_1_ = 36 dB and G_2_ = 24 dB; and (**e**) G_1_ = 42 dB and G_2_ = 18 dB.

A more detailed noise analysis of the noise signals vs. G_1_ gain is reported in Figure 6. Starting at 18 dB (Figure 6a), as the first stage gain increases, the contribution of the TDM decreases, leaving room for the electronic noise signal. Specifically, Figure 6 shows v_nTDM_, v_ne_ and total noise (v_ntot_, the quadratic sum of TDM artifact signal power and electronic noise) for G_1_ = 18 dB up to G_1_ = 42 dB with 6 dB/step.

As the first stage amplification value gradually increases, electronic noise becomes dominant and the TDM artifacts tend to be negligible (as in Figure 6e) where electronic noise practically overlaps the TDM contribution.

This trend would seem to suggest maximizing the gain of the first amplifying stage as much as possible compared to the second amplifier stage. Unfortunately, this solution would involve an excessive increase in both power and area requirements for the first stage, which is located exactly at the interface with the extracellular environment, significantly reducing the advantages of using the TDM technique.

**Figure 7:**
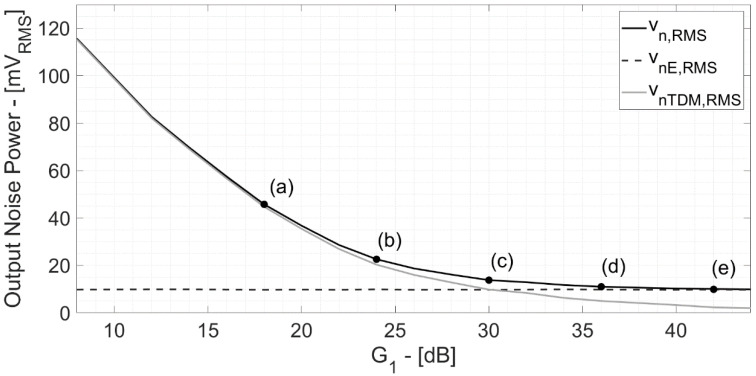
Components of the output noise power at the end of the second amplification stage vs. G_1_. Points (a)–(e) correspond to the same gain distributions of the time-domain simulations of Figure 6. The operating point (c) (G_1_ = 30 dB, G_2_ = 30 dB) occurs when the TDM artifact signal power becomes equal to the electronic noise contribution.

Figure 7 reports the extracted values of the total output noise power in the neural AFE vs. G_1_. There is a specific operating point threshold (G_1_ = 30 dB and G_2_ = 30 dB) in which the TDM artifact signal power becomes equal to the electronic counterpart.

**Figure 8:**
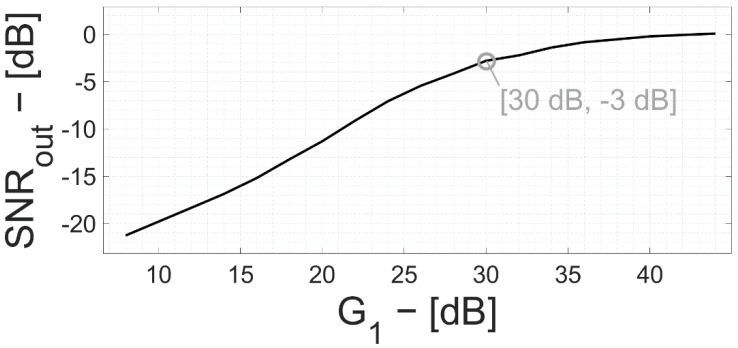
Signal-to-noise ratio at the input node of the a-to-d converter vs. gain G_1_, considering a signal of the same power of the electronic noise at the interface.

In Figure 8, the channel SNR is plotted vs. G_1_, assuming a neuro-potential signal power at the beginning of the chain equal to the electronic noise power (i.e., 10 µV_RMS_). The SNR is negative for small values of G_1_ and approaches 0 dB for G_1_ >> 30 dB.

Defining the noise factor F as the ratio between input SNR (SNR_in_, due to the only electronic noise) and the output SNR (SNR_out_, due to both electronic and TDM offset-sampling effect):

(7) $$F=\frac{{\mathrm{SNR}}_{\mathrm{in}}}{{\mathrm{SNR}}_{\mathrm{out}}}=\frac{{\left(\frac{v_{\mathrm{neuro},\mathrm{RMS}}}{v_{\mathrm{ne},\mathrm{RMS}}}\right)}^{2}}{\frac{{\left(v_{\mathrm{neuro},\mathrm{RMS}}\cdot G_{1}\cdot G_{2}\right)}^{2}}{{\left(v_{\mathrm{ne},\mathrm{RMS}}\cdot G_{1}\cdot G_{2}\right)}^{2}+{\left(v_{\mathrm{nTDM},\mathrm{RMS}}\cdot G_{2}\right)}^{2}}}=1+{\left(\frac{1}{G_{1}}\cdot \frac{v_{\mathrm{nTDM},\mathrm{RMS}}}{v_{\mathrm{ne},\mathrm{RMS}}}\right)}^{2}$$

then the channel noise figure (NF) is:

(8) $$\mathrm{NF}=10\cdot \log_{10}\left(F\right)$$

**Figure 9:**
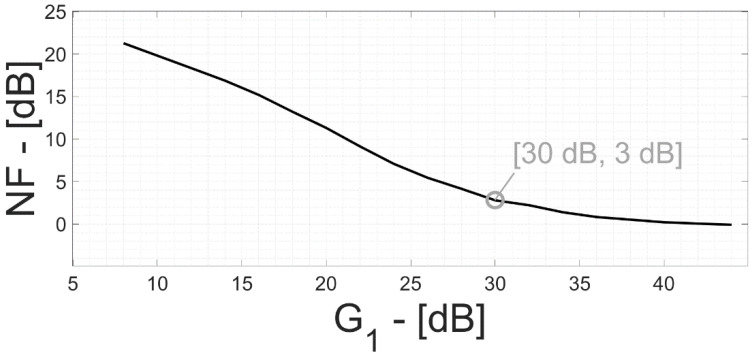
Noise figure of the acquisition channel and gain G_1_ of the first amplification stage considering a signal of the same power of the electronic noise at the interface.

NF vs. G_1_ is plotted in Figure 9, demonstrating that the SNR degradation reduces with G_1_ increasing. NF = 3 dB occurs at G_1_ = 30 dB; that is, when electronic noise and TDM artifact signal have the same power. Table 3 shows noise power, SNR and NF at different G_1_ (G_2_) values with a signal of the same power of the electronic noise at the interface.

**Figure 10:**
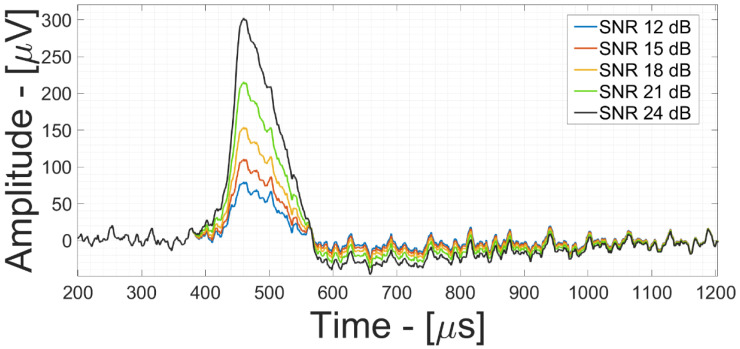
Action potentials with noise and TDM artifact contribution time-domain simulations referred to the input node of the neural AFE for different SNR_out_ values at G_1_ = 30 dB and G_2_ = 30 dB.

Figure 10 shows the electronic noise and TDM offset-sampling contribution time-domain simulation results (set at the optimum operating point threshold of G_1_ = 30 dB and G_2_ = 30 dB), comparing different AP signals having different SNR values (from 12 dB to 24 dB with 3 dB/step). This demonstrates that by efficiently calibrating the gain values of the amplification stages, it is possible to detect, by simple threshold crossing approach [23,24], weak extracellular neural events in the order of 50 µV_RMS_ with an SNR_out_ = 12 dB.

## 5. Conclusions

This paper presents a complete model of a time-division multiplexed acquisition channel for silicon–cell interfaces, which allow the minimization of the multiplexing artifacts introduced by the sampling operation and the maximization of the SNR at the a-to-d converter input. More specifically, with an electronic noise power of 10 µV_RMS_ at the interface and a TDM artifact signal power of 300 µV_RMS_, the front-end optimal operating point, G_1_ = 30 dB and G_2_ = 30 dB, as shown in Figure 10, exhibits an SNR of 12 dB for weak extracellular neural signals in the order of 50 µV_RMS_.

## Figures and Tables

**Table 1:** Neural AFE System-Level Model Design Parameters.

Parameter	Symbol	Value
1st Amplification Stage Low Frequency Gain	G_1_	0 dB–60 dB
1st Amplification Stage Dominant Pole Frequency	f_1_	7.5 kHz
2nd Amplification Stage Low Frequency Gain	G_2_	0 dB–60 dB
2nd Amplification Stage Dominant Pole Frequency	f_2_	1 MHz
TDM Sampling Frequency	f_TDM_	256 kHz
Max. Neuro-Potential Signal Frequency	f_0,neuro_	1 kHz
Over-Sampling Ratio	OVR	16
MEA Channel Pixel Count	N_pixel_	8

**Table 2:** Neural AFE Noise Model and TDM Artifact Design Parameters.

Parameter	Symbol	Value
Electronic Noise Source	v_ne_	-
Electronic Noise at Cell–Silicon Interface Power (1 Hz–5 kHz bandwidth)	v_ne,RMS_	10 µV_RMS_
Electronic Noise at Cell–Silicon Interface Power Spectral Density (1 Hz–5 kHz bandwidth)	<v^2^_ne_>/Δf	(141 nV/√Hz)^2^
TDM Artifact Source	v_nTDM_	-
TDM Artifact Signal Power (1 Hz–5 kHz bandwidth)	v_nTDM,RMS_	300 µV_RMS_
A-to-D Converter Number of Bits	Nb	10
A-to-D Converter Full Scale	FS	1 V
A-to-D Converter Quantization Noise Power	v_nADC,RMS_	281 µV_RMS_

**Table 3:** Neural AFE simulation results. SNR_out_ is calculated considering a signal of the same power of the electronic noise at the interface.

G_1_ [dB]	G_2_ [dB]	V_n,RMS,out_ [mV_RMS_]	V_nTDM,RMS,out_ [mV_RMS_]	SNR_out_ [dB]	NF [dB]
18	42	45.7	44.7	−13.2	13.2
24	36	22.6	20.3	−7.1	7.1
30	30	13.8	9.8	−3.0	3.0
36	24	11.0	5	−0.8	0.8
42	18	9.9	2.3	−0.06	0.06

## Data Availability

The data presented in this study are available on request from the corresponding author.
